# Buprenorphine Use Trends Following Removal of Prior Authorization Policies for the Treatment of Opioid Use Disorder in 2 State Medicaid Programs

**DOI:** 10.1001/jamahealthforum.2022.1757

**Published:** 2022-06-24

**Authors:** Shailina Keshwani, Michael Maguire, Amie Goodin, Wei-Hsuan Lo-Ciganic, Debbie L. Wilson, Juan M. Hincapie-Castillo

**Affiliations:** 1 Department of Pharmaceutical Outcomes and Policy, College of Pharmacy, University of Florida, Gainesville; 2 Center for Drug Evaluation and Safety, College of Pharmacy, University of Florida, Gainesville; 3 Department of Epidemiology, Gillings School of Global Public Health, University of North Carolina at Chapel Hill; 4 Injury Prevention Research Center, University of North Carolina at Chapel Hill

## Abstract

**Importance:**

State Medicaid programs have implemented initiatives to expand treatment coverage for opioid use disorder (OUD); however, some Medicaid programs still require prior authorizations (PAs) for filling buprenorphine prescriptions.

**Objective:**

To evaluate the changes in buprenorphine use for OUD among Medicaid enrollees in states that completely removed buprenorphine PA requirements.

**Design, Setting, and Participants:**

This retrospective cross-sectional study analyzed the immediate and trend changes on buprenorphine use during 2013 to 2020 associated with removal of PA requirements using a controlled interrupted time series analysis to account for autocorrelation. Data were collected from Medicaid State Drug Utilization Data for 2 states (California and Illinois) that completely removed a buprenorphine PA during the study period, and buprenorphine prescriptions for OUD treatment were identified among Medicaid enrollees.

**Main Outcomes and Measures:**

Quarterly total number of buprenorphine prescriptions for each state was calculated, and stratification analyses were conducted by dosage form (films and tablets).

**Results:**

Among the 2 state Medicaid programs (California and Illinois) that removed buprenorphine PAs, there was a total of 702 643 and 415 115 eligible buprenorphine prescription claims, respectively. After removing PA requirements for buprenorphine, there was an immediate increase that was not statistically significant (rate ratio [RR], 1.11; 95% CI, 0.76-1.61) in the number of all buprenorphine prescriptions in California and a statistically significant increase (RR, 6.99; 95% CI, 4.67-10.47) in the number of all buprenorphine prescriptions in Illinois relative to the change in the control states (Alabama, Florida, Idaho, Kansas, Mississippi, Nevada, South Dakota, and Wyoming). Additionally, there was a statistically significant decreasing trend in the number of all buprenorphine prescriptions in California (RR, 0.88; 95% CI, 0.82-0.94) and a statistically significant increasing trend in Illinois (RR, 1.11; 95% CI, 1.05-1.19) relative to the trend in control states.

**Conclusions and Relevance:**

In this cross-sectional study, removal of buprenorphine PA requirements was associated with a statistically significant increase in the number of buprenorphine prescription fills among Medicaid populations in 1 of the 2 included states.

## Key Points

**Question:**

What are the outcomes of removing prior authorizations (PAs) on buprenorphine for opioid use disorder (OUD) in Medicaid programs?

**Findings:**

In this cross-sectional study of Medicaid State Drug Utilization Data from 2 states, buprenorphine prescribing for OUD in the state Medicaid program of Illinois increased after removing PA requirements, whereas a similar increase was not observed in the state Medicaid program of California.

**Meaning:**

Policies removing PA requirements for buprenorphine prescribing for treatment of OUD may improve access to OUD treatment in Medicaid enrollees.

## Teaser

This cross-sectional study evaluates the changes in buprenorphine use for opioid use disorder among Medicaid enrollees in states that completely removed buprenorphine prior authorization requirements.

## Introduction

An estimated 1.4 million individuals in the US had a diagnosis of opioid use disorder (OUD) in 2019.^1^ Despite clinical guidelines and supporting evidence^2,3,4^ for the effectiveness and safety of medication treatment for OUD (MOUD), fewer than 35% to 58% of adults diagnosed with OUD in the US received treatment according to 2019 estimates.^5,6^ Buprenorphine and buprenorphine-naloxone combination products (hereafter buprenorphine for OUD) are associated with decreased opioid-related overdoses and infectious disease transmission.^7^

Unlike methadone, which is administered through opioid treatment programs, prescribers with waivers can prescribe buprenorphine for OUD in office-based settings. However, some government-sponsored insurance and commercial insurance plans require prior authorizations (PAs) before a patient can fill a buprenorphine prescription for OUD. Currently, Medicaid preferred drug lists (PDLs) with PAs for buprenorphine for OUD are a cost-control tool, and PAs have been used by commercial insurers in negotiation of rebates to reduce expenditures.^8^ These PAs vary considerably and may include variations by PDL status of each product, step therapy requirements, quantity limits on daily dose, lifetime treatment limits,^9,10^ and physician documentation indicating patient compliance to buprenorphine counseling requirements.^11^ The PA policies in single-state Medicaid programs have been associated with reductions in buprenorphine doses more than 24 mg per day (the maximum recommended therapeutic dose)^12^ and lower rates of abuse and overdose among Medicaid enrollees.^13^ These studies were conducted before Medicaid expansion took off in 2014—many state Medicaid programs have implemented new PA policies related to buprenorphine for OUD in more recent years. Recent studies have reported that removal of PAs has been associated with improved health outcomes (eg, fewer emergency department visits and hospitalizations) in Medicare enrollees.^14,15^

Buprenorphine PA requirements in Medicaid programs have been rated by health care providers as the highest barrier to accessing MOUD,^16^ creating delays or interruptions in prescription fills, and require coordinating authorization requests^17^ between prescribers, payers, and pharmacies. These barriers can lead to serious consequences for individuals with OUD, including relapse,^18^ other illicit drug use,^19^ or death from overdose.^20^ Some Medicaid programs have taken initiatives to expand MOUD coverage by lifting buprenorphine PA restrictions.^21^ Given that Medicaid enrollees have disproportionately higher risks for both OUD and overdose,^22^ we aimed to evaluate the changes in use of buprenorphine for OUD among Medicaid enrollees in states that completely removed PA policies for buprenorphine prescribing. We hypothesized that completely eliminating buprenorphine PA requirements for OUD treatment would result in immediate and sustained increases in buprenorphine prescribing among Medicaid enrollees.

## Methods

### Study Design

This retrospective repeated cross-sectional study used controlled interrupted time series (CITS) models to evaluate trend and level changes associated with complete removal of PA requirements in state Medicaid programs in 2 states: California and Illinois. The University of Florida Institutional Review Board approved this study, which was exempt from informed consent requirements because data were deidentified. The study adheres to the Strengthening the Reporting of Observational Studies in Epidemiology (STROBE) reporting guideline for cross-sectional studies.

### Data Sources

We used publicly available Medicaid State Drug Utilization Data to analyze pharmacy prescription claims for buprenorphine and its combination products with indications for OUD from the fourth quarter of 2013 to the first quarter of 2020. Each state reports drug utilization data for outpatient medications covered under the Medicaid Drug Rebate Program,^23^ and this is inclusive of prescription claims for both Medicaid and Children’s Health Insurance Program (CHIP) enrollees.^23^ These data are deidentified, and aggregated variables of dispensed medications include number of prescriptions, drug name, National Drug Code (NDC), a plan type indicator (ie, fee-for-service or managed care), and a data suppression indicator. The suppression indicator identifies suppressed records for each specific drug product or NDC having a number of prescriptions dispensed fewer than 11 times within the respective state program in any given quarter. The quarterly total state Medicaid and CHIP enrollment was measured using 2 data sources. For the fourth quarter of 2013, we used monthly Medicaid and CHIP enrollment data provided by the Centers for Medicare & Medicaid Services for December 2013.^24^ Beginning in 2014, we used Medicaid enrollment reports from the Kaiser Family Foundation, which included aggregated Medicaid and CHIP enrollment data for each state for January 2014 through November 2020.^25^ This source provides monthly enrollment counts; therefore, we used the last month of each quarter as the denominator for the entire quarter from 2014 onward.

### Operationalization of Policy Interruption and Selection of Study Population

We searched published literature and state registers (legislation, regulatory, and administrative actions) for policies related to PAs for medications for substance use disorder treatments. Identified in this search, reports from Miller^26^ and Weber^21^ were used as preliminary guides to narrow which states had potentially relevant Medicaid PA policies. After identifying these states and policies, we searched the primary sources from 2013 onward, then categorized each state as either having strict PA requirements or having policies implemented for removing PAs related to buprenorphine for OUD. Primary policy sources included state registers as well as documents related to Medicaid rulemaking. For the states that implemented, revised, or removed relevant PA policies, we recorded their effective dates, policy type, and policy change description. We also recorded exemptions related to buprenorphine dosages and formulations, and if available, we reviewed and extracted information on buprenorphine products included on the PDL for each Medicaid program (eTable 3 in the Supplement).

We required included study states to have at least 7 data points before and after the intervention^27^ for CITS analyses. After comprehensive policy surveillance and assessment, Medicaid prescription data from California and Illinois were included in the analyses because these states had completely removed buprenorphine PAs during the study period (ie, 2013-2020), and each state had sufficient prescription data points available to evaluate prescription changes before and after policy implementation. Other states such as Colorado, New Jersey, and Texas also implemented such policies^21^; however, the implementation period of these state policies did not allow sufficient data points to evaluate changes after the intervention using the quarter-level data (eg, Texas implemented HB 2174 effective from September 1, 2019, prohibiting Medicaid from requiring PAs for medications for OUD).^28^ We excluded states that had partial removal of PAs (eg, Utah does not require PAs for buprenorphine for OUD for the first 180 days of treatment, but to continue the treatment prescribers must provide detailed attested documents justifying the need to continue the treatment).^26^

### Outcome Measure

We used NDCs to identify buprenorphine and its combination products for OUD and their dosage forms (ie, film, tablet, patch, injection solution). We excluded transdermal patch and injection solution dosage forms that are exclusively indicated for pain management.^29,30,31,32^ There were no records for intradermal implant products in the present data. The outcome variable was calculated as the total number of buprenorphine prescriptions using Medicaid enrollees by quarter as an offset (ie, total number of buprenorphine prescriptions each quarter divided by total number of Medicaid enrollees in the last month of each quarter). To further characterize the utilization of buprenorphine for OUD, we conducted stratification analyses by dosage form (ie, films and tablets) for all prescription claims.

### Statistical Analysis

We analyzed quarterly buprenorphine utilization using CITS models accounting for autocorrelation of error terms. We analyzed trends before policy implementation (ie, time effect) and both immediate changes (ie, level effect) and trend changes (ie, trend effect) in the outcome after implementation compared with the control. We included a 2-quarter phase-in period after the intervention to accommodate for the time lag from initial introduction of policy to its full effect. Each state implemented policies removing PAs related to buprenorphine prescribing in different months during the study period; therefore, interruption is state specific. Because we used quarterly data and policy was implemented in a particular month, we defined the interruption in the quarter of that month (eg, for June 1, 2015, the interruption was defined as second quarter of 2015).

To control for secular trends in buprenorphine use and to account for federal-level policy that could affect drug utilization trends across the included states, we adjusted the models using a control.^33^ We identified 8 states that did not remove PAs for the preferred buprenorphine products in their Medicaid programs. We aggregated pharmacy prescription claims and Medicaid enrollment data from these 8 states (Alabama, Florida, Idaho, Kansas, Mississippi, Nevada, South Dakota, and Wyoming) to construct the control (eTables 2 and 4 in the Supplement).

We fitted generalized linear models with log link for all analyses. We observed overdispersion in the data and therefore conducted goodness-of-fit tests to decide whether to use a Poisson or negative binomial model. If required, we adjusted for autoregressive moving average correlation structure of order (p, q) by visually inspecting autocorrelation function and partial autocorrelation function plots and compared Akaike information criteria to obtain the best model for the data. Data management and analysis were conducted using R, version 4.0.3 (R Project for Statistical Computing), and SAS, version 9.4 (SAS institute Inc).

### Sensitivity Analysis

Because policies implemented under each state Medicaid program required the changes in regulations or law, the complete effect of these policies may not be seen immediately after the effective date. Therefore, we conducted several sensitivity analyses to ensure the robustness of the findings by introducing different phase-in periods: (1) no phase-in period, (2) 1-quarter phase-in period, and (3) 3-quarter phase-in period after the implementation of policies removing PAs related to buprenorphine. In addition, we assessed the robustness of results based on the selection of control states by reanalyzing the data for California and Illinois using the synthetic control method that gives different weights to control states and variables (eMethods in the Supplement).

## Results

### Policies Identified

California implemented a policy change within the Medi-Cal program to remove the PA requirement starting June 1, 2015, for either buprenorphine or buprenorphine-naloxone formulations when prescribed by qualified physicians for OUD treatment.^34,35^ On the other hand, the legislature of Illinois prohibited PAs for all US Food and Drug Administration–approved forms of MOUD effective from July 1, 2015^26,36^ (eTable 1 in the Supplement).

### Trends in Use of Buprenorphine Products by States

#### California

**Figure 1.**
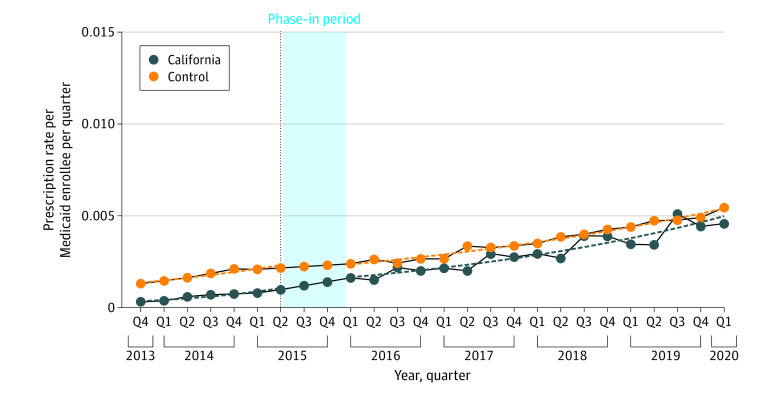
Trends in Utilization of Buprenorphine Prescriptions by Quarter for the State Medicaid Program of California vs Control Dashed lines represent the fitted model.

**Figure 2.**
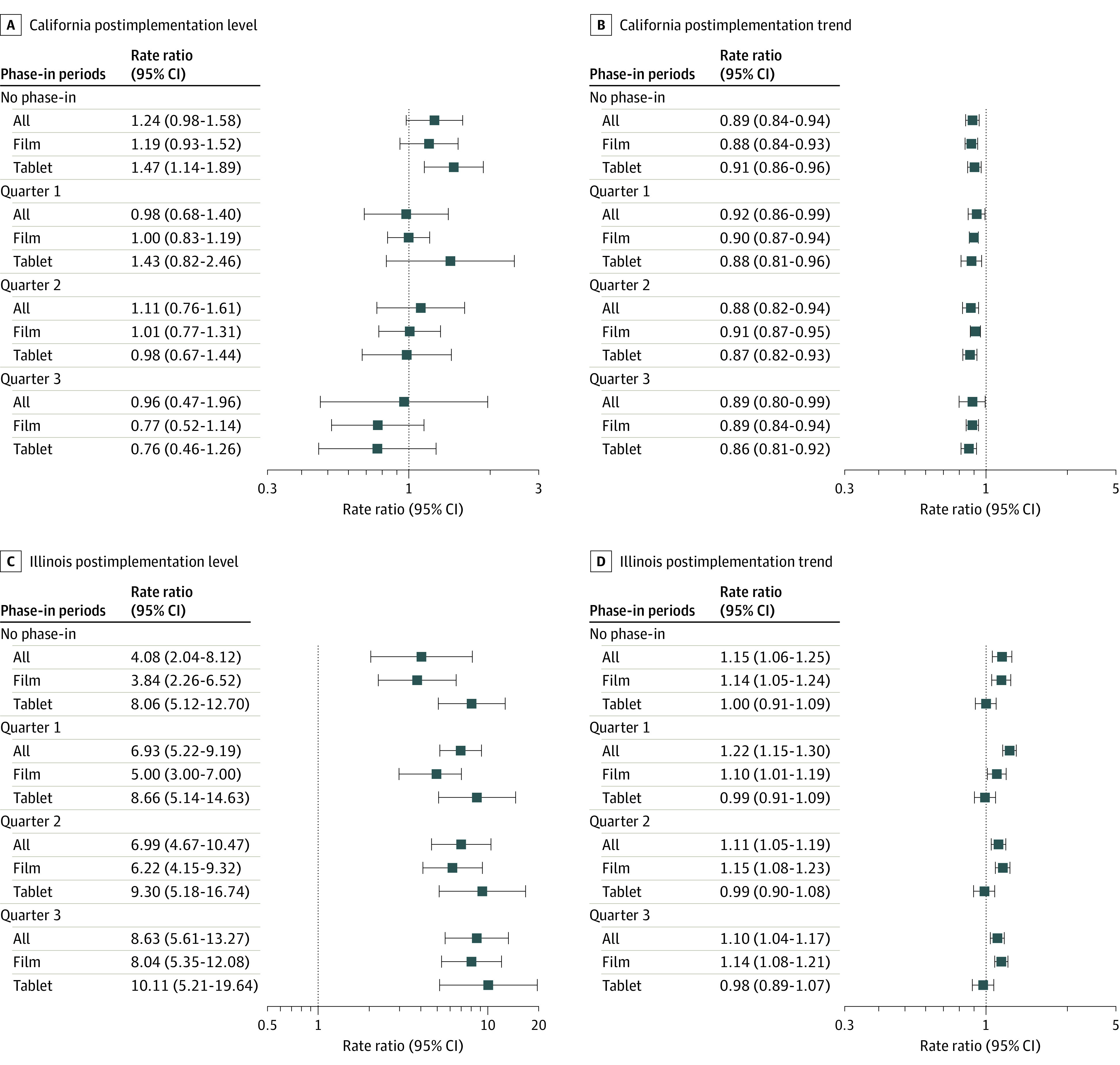
Trends in Utilization of Buprenorphine Prescriptions by Different Phase-in Periods and Dosage Forms

After the removal of PA requirements in California, there was an immediate increase that was not statistically significant (rate ratio [RR], 1.11; 95% CI, 0.76-1.61) in the number of all buprenorphine prescriptions relative to the change in the control and a relative statistically significant decreasing trend (RR, 0.88; 95% CI, 0.82-0.94) in the number of all buprenorphine prescriptions (Table and Figure 1). When stratifying analyses by dosage form, the policy change was associated with an immediate increase that was not statistically significant (RR, 1.01; 95% CI, 0.77-1.31) in the number of prescriptions for films relative to the change in the control, as well as a relative decrease that was not statistically significant (RR, 0.93; 95% CI, 0.67-1.44) in the number of tablets (Table and Figure 2A and B).

**Table.** Coefficients From Controlled Interrupted Times Series Models Using Autoregressive Moving Averages (Quarterly) for Buprenorphine Utilization With 2-Quarter Phase-in Periods

State	Dosage form	Rate ratio (95% CI)		
		Preimplementation trend	Postimplementation level change	Postimplementation trend change
California	All	1.15 (1.08-1.23)	1.11 (0.76-1.61)	0.88 (0.82-0.94)
	Film	1.13 (1.09-1.17)	1.01 (0.77-1.31)	0.91 (0.87-0.95)
	Tablet	1.16 (1.10-1.23)	0.93 (0.67-1.44)	0.87 (0.82-0.93)
Illinois	All	0.90 (0.85-0.96)	6.99 (4.67-10.47)	1.11 (1.05-1.19)
	Film	0.88 (0.83-0.93)	6.22 (4.15-9.32)	1.15 (1.08-1.23)
	Tablet	1.01 (0.93-1.10)	9.30 (5.18-16.74)	0.99 (0.90-1.08)

#### Illinois

**Figure 3.**
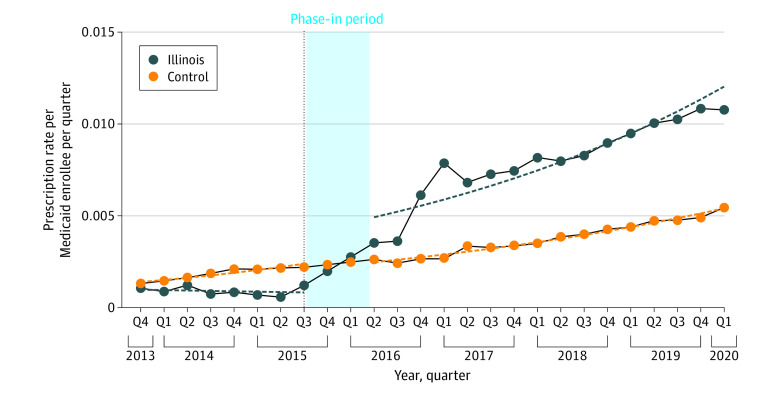
Trends in Utilization of Buprenorphine Prescriptions by Quarter for the State Medicaid Program of Illinois vs Control Dashed lines represent the fitted model.

After PA removal in Illinois, there was a statistically significant immediate increase (RR, 6.99; 95% CI, 4.67-10.47) in the number of all buprenorphine prescriptions relative to the change in the control, with a relative statistically significant increasing trend in the number of all buprenorphine prescriptions (RR, 1.11; 95% CI, 1.05-1.19) (Table and Figure 3). Similarly, there was a statistically significant immediate relative increase in the number of prescriptions for films (RR, 6.22; 95% CI, 4.15-9.32) and tablets (RR, 9.30; 95% CI, 5.18-16.74) in the postimplementation phase with a relative statistically significant increasing trend in the number of films (RR, 1.15; 95% CI, 1.08-1.23) and a relative decreasing trend that was not statistically significant in the number of tablets (RR, 0.99; 95% CI, 0.90-1.08) (Table and Figure 2C and D).

### Sensitivity Analysis

Sensitivity analyses using different phase-in definitions yielded similar results as the primary analysis (Figure 2). There was a decrease that was not statistically significant in the number of all buprenorphine prescriptions (ie, level change) in California with 1-quarter and 3-quarter phase-in periods (Figure 2), in contrast with an increase that was not statistically significant in the primary analysis. Results using the synthetic control method aligned with the main findings in identifying a gap in the buprenorphine prescribing rate in Illinois but not in California (eFigures 1-8 in the Supplement).

## Discussion

Although both California and Illinois implemented complete removal of PA policies for buprenorphine for OUD, we observed heterogeneity in the findings between these 2 states. In the state Medicaid program of California, we observed an immediate increase that was not statistically significant in the number (ie, level change) of all buprenorphine prescriptions for OUD with a statistically significant decreasing trend after the removal of PA requirements. In contrast, a statistically significant immediate increase in level and trend was observed in the state Medicaid program of Illinois after the legislative changes in PAs for medication-assisted treatments. These different findings may be explained by the strict lifetime caps and rigid tapering dosage for the treatment of OUD with buprenorphine that existed in Illinois prior to the policy change,^11,37^ whereas no such requirements existed in California.^38^

The trend in buprenorphine prescribing in California slowed down considerably compared with the prepolicy period. This statistically significant decrease in trend during the postpolicy period is, thus, not to be interpreted as a reversal of the baseline increased utilization but rather a decrease in the rate of prescribing. In California, prescribing of buprenorphine for OUD was already increasing substantially before 2015; thus, the effect of removal of PA for Medicaid enrollees was not as pronounced in the postimplementation period. A recent study that supports these findings showed that Medicaid MOUD prescribing far outpaced non-Medicaid MOUD prescribing from 2012 to 2014 in California.^39^ Also, previous studies have reported that methadone is more commonly used among Medicaid enrollees in California than buprenorphine.^35^ In addition, the PA procedure in California may not be a substantial administrative barrier compared with that of Illinois—California uses a single, standardized form for all PAs regardless of medication type, and PA requests are accepted from any delivery means.^40^

New formulations of buprenorphine products were added or removed from each state’s respective PDL throughout the study period. These PDL changes could also influence observed use patterns, as could other policies that were implemented at the federal level. One such policy, the 2016 Comprehensive Addiction and Recovery Act, expanded buprenorphine prescribing authority in office-based settings to qualified nurse practitioners and physician assistants, who were then eligible to acquire buprenorphine prescribing waivers in 2017.^41^ Some individual states also passed versions of provisions found within the Comprehensive Addiction and Recovery Act at different times (eg, restricting nurse practitioners with waivers from prescribing medications for OUD treatment).^42^ Such policy and/or law changes may have influenced the trend in buprenorphine prescribing during the study period but because this study was conducted using a control to account for such federal policy and/or law changes, the observed effect estimates may have not been influenced to some extent.

Most states also include naltrexone or methadone in the PDL, and services such as counseling, rehabilitation services, and psychological therapy may also be included as qualifying for OUD treatment coverage. Therefore, uptake of OUD treatments are likely dependent on a host of factors, including but not limited to prescriber specialty, prescriber waiver status, clinician’s willingness and treatment preferences, concerns related to patient diversion, and PA requirements.^43^ Previous studies reported that most waivered clinicians prescribe buprenorphine below their capped number of patients,^44^ and few prescribe close to the cap.^45^ Previous studies indicate that 19% to 47% of surveyed practices that provide buprenorphine treatment for OUD only accept cash-only payments,^46^ which suggests that Medicaid enrollees have limited access. Lastly, national estimates further demonstrate amplified disparities in access to buprenorphine and other MOUD among racial and ethnic minorities.^47^ Further research is required to quantify buprenorphine prescribing initiation and continuity within the Medicaid population and to connect it with patient health outcomes.

### Limitations

First, Medicaid State Drug Utilization Data lacks information about indications for corresponding prescriptions claims. However, the present analyses focused on buprenorphine products approved by the US Food and Drug Administration for OUD. Second, more so than state and federal statutes, state Medicaid policies from rulemaking and administrative action may take longer to disseminate; thus, findings that were not statistically significant might reflect a delay in policy implementation and not a lack of an effect. We conducted the primary analysis with a 2-quarter phase-in period to account for these delays and included sensitivity analyses with no phase-in, 1-quarter phase-in, and 3-quarter phase-in periods. Overall, the results were robust across these sensitivity analyses, which included a complete reanalysis of the data using the synthetic control method. The latter methodology allows for explicit specification of contributing variables for each control state to best resemble the intervention states.^48,49^ Third, the data included in this study did not distinguish beneficiaries between Medicaid and CHIP, given the aggregated nature of the prescription claims in public files. Future studies should stratify analyses by Medicaid eligibility criteria and other patient demographics to answer questions related to equity of care and effects of policy for different Medicaid populations. Fourth, we acknowledge that other policies and trends may not be sufficiently captured or controlled for even with the inclusion of a control group. For a program-wide policy change in a large payer like Medicaid, we argue that analyzing aggregated drug utilization data provides an informative and straightforward indicator for policy makers as a first step in a comprehensive policy evaluation. Additional research should account for individual level heterogeneity using clinical and socioeconomic variables. Finally, the findings from 2 state Medicaid programs may not be generalizable to other state Medicaid programs or other populations. Nevertheless, these findings can inform other state Medicaid programs and policy makers on the potential effects associated with the removal of PA requirements for their patient population.

## Conclusions

In this cross-sectional study using Medicaid State Drug Utilization Data from 2013 to 2020, findings indicate that complete removal of PA requirements for buprenorphine prescriptions for OUD was associated with a statistically significant increase in the number of buprenorphine prescription fills among Medicaid populations in Illinois. A similar increase was not observed among the Medicaid population in California. The heterogeneity in these findings might indicate that buprenorphine prescribing may not only depend on PA policies, but also other factors such as prepolicy utilization trends.

## Notes

Supplement. **eTable 1.** Policies for States Removing Buprenorphine Prior Authorization Requirements in Medicaid (Intervention States) **eTable 2.** Prior Authorization Policies Related to Buprenorphine for OUD in Control States **eTable 3.** Legal Mapping for State Medicaid Prior Authorization Policies Related to Buprenorphine for OUD **eTable 4.** Analytical Dataset. Source: State Drug Utilization Data **eMethods.** Sensitivity Analyses: Synthetic Control Method **eFigure 1.** States Weights and Variable Weights in the Synthetic California **eFigure 2.** States Weights and Variable Weights in the Synthetic Illinois **eFigure 3.** Trends in Buprenorphine Prescribing Rate in California vs. Synthetic California **eFigure 4. **Buprenorphine Prescribing Rate Gap in California vs. Synthetic California **eFigure 5.** Trends in Buprenorphine Prescribing Rate in Illinois vs. Synthetic Illinois **eFigure 6.** Buprenorphine Prescribing Rate Gap in Illinois vs. Synthetic Illinois **eFigure 7.** Ratio of Post-Intervention RMSPE to Pre-Intervention RMSPE: California and Control States **eFigure 8.** Ratio of Post-Intervention RMSPE to Pre-Intervention RMSPE: Illinois and Control States
eReferences
