# Supplementary material for: Buprenorphine Use Trends Following Removal of Prior Authorization Policies for the Treatment of Opioid Use Disorder in 2 State Medicaid Programs
Source: JAMA Health Forum. 2022 Jun 24;3(6):e221757. doi: 10.1001/jamahealthforum.2022.1757 (PMC9233239; doi:10.1001/jamahealthforum.2022.1757)
Supplement: Supplement. — eTable 1. Policies for States Removing Buprenorphine Prior Authorization Requirements in Medicaid (Intervention States) eTable 2. Prior Authorization Policies Related to Buprenorphine for OUD in Control States eTable 3. Legal Mapping for State Medicaid Prior Authorization Policies Related to Buprenorphine for OUD eTable 4. Analytical Dataset. Source: State Drug Utilization Data eMethods. Sensitivity Analyses: Synthetic Control Method eFigure 1. States Weights and Variable Weights in the Synthetic California eFigure 2. States Weights and Variable Weights in the Synthetic Illinois eFigure 3. Trends in Buprenorphine Prescribing Rate in California vs. Synthetic California eFigure 4. Buprenorphine Prescribing Rate Gap in California vs. Synthetic California eFigure 5. Trends in Buprenorphine Prescribing Rate in Illinois vs. Synthetic Illinois eFigure 6. Buprenorphine Prescribing Rate Gap in Illinois vs. Synthetic Illinois eFigure 7. Ratio of Post-Intervention RMSPE to Pre-Intervention RMSPE: California and Control States eFigure 8. Ratio of Post-Intervention RMSPE to Pre-Intervention RMSPE: Illinois and Control States eReferences [file jamahealthforum-e221757-s001.pdf]

Supplemental Online Content

Keshwani S, Maguire M, Goodin A, Lo-Ciganic WH, Wilson DL, Hincapie-Castillo JM. Buprenorphine use trends following removal of prior authorization policies for the treatment of opioid use disorder in 2 state Medicaid programs. *JAMA Health Forum*. 2022;3(6):e221757. doi:10.1001/jamahealthforum.2022.1757

eTable 1. Policies for States Removing Buprenorphine Prior Authorization Requirements in Medicaid (Intervention States)

eTable 2. Prior Authorization Policies Related to Buprenorphine for OUD in Control States

eTable 3. Legal Mapping for State Medicaid Prior Authorization Policies Related to Buprenorphine for OUD

eTable 4. Analytical Dataset. Source: State Drug Utilization Data

eMethods. Sensitivity Analyses: Synthetic Control Method

eFigure 1. States Weights and Variable Weights in the Synthetic California

eFigure 2. States Weights and Variable Weights in the Synthetic Illinois

eFigure 3. Trends in Buprenorphine Prescribing Rate in California vs. Synthetic California

eFigure 4. Buprenorphine Prescribing Rate Gap in California vs. Synthetic California

eFigure 5. Trends in Buprenorphine Prescribing Rate in Illinois vs. Synthetic Illinois

eFigure 6. Buprenorphine Prescribing Rate Gap in Illinois vs. Synthetic Illinois

eFigure 7. Ratio of Post-Intervention RMSPE to Pre-Intervention RMSPE: California and Control States

eFigure 8. Ratio of Post-Intervention RMSPE to Pre-Intervention RMSPE: Illinois and Control States

eReferences

This supplemental material has been provided by the authors to give readers additional information about their work.

© 2022 Keshwani S et al. *JAMA Health Forum.*

eTable 1. Policies for States Removing Buprenorphine Prior Authorization Requirements in Medicaid (Intervention States)

State	Effective date for policies	Description	Exemptions
California	June 1, 2015	PA request is not required by Medi-Cal for buprenorphine-containing products when prescribed by qualified physicians for the treatment of individuals with opioid addiction.[1,2]	See document [3] for specific medications that does not require PA since 2019.
Illinois	July 1, 2015	FDA approved medication assisted treatment for opioid dependence is covered under both fee for service and managed care medical assistance programs and do not require PA mandate, or lifetime restriction limit mandate.[3,4]	N/A

PA- prior authorizations, PDL-preferred drug list, OUD- opioid use disorder

© 2022 Keshwani S et al. *JAMA Health Forum.*

eTable 2. Prior Authorization Policies Related to Buprenorphine for OUD in Control States

State	Description	Last updated
Alabama	PA is required for medication treatment for OUD, which includes buprenorphine.[5,3]	November 2019
Florida	PA is required for all buprenorphine formulations. 7 days' supply of buprenorphine formulations is allowed for patients who have not undergone MAT in the last 12 months without PA for induction with preferred medications, with a maximum of two 7 days induction refills within 60 days. After induction PA is required.[3]	October 2019
Idaho	PA is required for preferred drugs that include Suboxone, and generic buprenorphine/naloxone.[3,6]	October 2019<br>January 2022
Kansas	PA requiring documentation of clinical criteria and participation in behavioral treatment. No approved medication OUD is listed under the PDL.[3]	October 2019
Mississippi	Prior authorization is required for preferred and non-preferred drugs. Suboxone and generic buprenorphine/naloxone are preferred that require patients to meet clinical pre-authorization criteria.[3]	November 2019
Nevada	Preferred buprenorphine formulations require PA.[3,7]	October 2019
South Dakota	PA is required for preferred and non-preferred buprenorphine formulations. It requires patients to meet clinical criteria, approval request for starting dose, regular drug test among other requirements.[3]	October 2019
Wyoming	Preferred and non-preferred buprenorphine formulations require PA.[3]	October 2019

PA- prior authorizations, PDL-preferred drug list, OUD- opioid use disorder, MAT- Medication Assisted Treatments

© 2022 Keshwani S et al. *JAMA Health Forum.*

eTable 3. Legal Mapping for State Medicaid Prior Authorization Policies Related to Buprenorphine for OUD

State	Complete Removal of PA	Partial Removal of PA	Effective Date of Removal	Description	Last Updated
Alaska	No	Yes	April 20, 2017	PA is not required for buprenorphine-based products used for MAT for the first 28-day fill. Products containing buprenorphine as single ingredient for MAT is not authorized for males.[8,3]	October 2019
Arizona	No	Yes	Unknown	PA is not required for preferred buprenorphine products, but it is required for non-preferred buprenorphine formulations.[3]	October 2019
Arkansas	No	Yes	April 12, 2019	PA is not required for preferred buprenorphine products. The state law was effective in April 12, 2019 but Medicaid program had until Jan1, 2020 to comply.[9]	June 2020
Colorado	No	Yes	January 1, 2020	Medicaid managed care plans do not require PA for any FDA approved medications for SUD covered by the plan.[10]	
Connecticut	Unknown	Unknown	Unknown	Preferred and no-preferred drug do not require PA[3]	August 2019
Delaware	No	Yes	January 1, 2017	Delaware Medicaid removed PA requirements for preferred drugs for treatment of OUD.[3] Non-preferred agents require a PA. See PDL [11] for list of preferred and non-preferred agents.	April 2017
District of Columbia	Yes	Yes	April-01-2019	All FDA approved medication for OUD prescribed and dispensed with PA in does up to 24mg/day [12]	April 2019
Georgia	No	Yes	November 2017	Fee-for-service Medicaid removed the PA requirement for preferred buprenorphine products in 2017 [13]. The PDL contains buprenorphine sublingual tablets and quantity limits may apply for a branded product.[3] Non-preferred medications that require PA include generic buprenorphine/naloxone SL tablets and others (quantity limits may apply).See document [3] for list of preferred and non-preferred agents.	October 2019
Hawaii	No	Yes	2012	PA is not required for most drugs used for the treatment of addiction[3]	November 2019
Indiana	No	Yes	-	PA is required for initial 6 months with renewal every 6 months[3]	September 2019
Iowa	Unknown	Unknown	June 1, 2019	At least one form of methadone, buprenorphine, naloxone, buprenorphine/ naloxone, and naltrexone can be prescribed without PA[14]	May 2019

© 2022 Keshwani S et al. *JAMA Health Forum.*

Kentucky	No	Yes	Unknown	PA is not required for preferred buprenorphine products, but it is required for non-preferred buprenorphine formulations.[3]	October 2019
Louisiana	No	Yes	2019	PA is not required for Buprenorphine/naloxone sublingual films.[15]	January 2020
Maine	No	Yes	June 16, 2020	PA is prohibited for at least one drug in each therapeutic class of medication used for MAT for OUD and for any MAT for OUD for pregnant women.[16]	May 2020
Maryland	No	Yes	-	PA is not required but quantity limits are applied.[3]	July 2019
Massachusetts	No	Yes	-	Preferred buprenorphine products do not require PA but all non-preferred formulations require PA[3]	October 2019
Michigan	No	Yes	-	PA is not required for 2 weeks supply for preferred buprenorphine products but after the initial period PA is required.[3]	December 2019
Minnesota	No	Yes	-	PA is not required for preferred buprenorphine products, but it is required for non-preferred buprenorphine formulations.[3]	July 2019
Missouri	Unknown	Unknown	July 11, 2019	PA is required for non-preferred buprenorphine products[3]. SB 514 was passed on July 11 2014 to include all current and new FDA approved formulation and medications for OUD.[16]	October 2019
Montana	Unknown	Unknown	-	Preferred and non-preferred formulations may require PA.[3]	September 2019
Nebraska	No	Yes	-	PA is required for non-preferred drugs which includes sublingual tablets.[3]	October 2019
New Hampshire	No	Yes	-	Certain Preferred and non-preferred medications for OUD do not require PA but quantity limits applied. (See PDL for more information)[17]	March 2022
New Jersey	Yes	Yes	October 15, 2019	FDA approved medications for OUD are covered without PA[18]	July 2019
New Mexico	No	Yes	-	Preferred buprenorphine formulations may not require PA, but non-preferred formulations require PA. The PDL is different for each Managed care plans. [3]	October 2019
New York*	No	Yes	August 2016	PA is not required for preferred buprenorphine products with quantity limits[3]	October 2019
North Dakota	No	No	-	Pre-authorization is required for preferred and non-preferred buprenorphine formulations.[3]	October 2019
Ohio	No	Yes	-	Preferred buprenorphine products do not require PA but all non-preferred formulations require PA[3]	October 2019

© 2022 Keshwani S et al. *JAMA Health Forum.*

Oklahoma	No	Yes	-	Preferred buprenorphine products do not require PA but all non-preferred formulations require PA.[3,19]	2018
Oregon	No	Yes	August 23, 2019	PA is not required for the first 30 days of MAT for OUD.[20]	2019
Pennsylvania	No	Yes	-	Preferred buprenorphine products do not require PA but all non-preferred formulations require PA[3]	October 2019
Rhode Island	Unknown	Unknown	-	Preferred buprenorphine products do not require PA.[3]	June 2018
South Carolina	No	Yes	-	Certain buprenorphine formulations do not require PA. [3]	July 2019
Tennessee	No	No	-	Preferred and non-preferred medications for OUD require PA. [3] Buprenorphine Enhanced Medication Assisted Recovery and Treatment (BESMART) Program was developed in 2019 to provide office based buprenorphine treatment.[21]	2019
Texas	Yes	Yes	September 1, 2019	PA is not required for MAT for OUD[16]	May 2020
Utah	No	Yes	-	PA is not required for certain buprenorphine formulations for the first 180 days but if treatment is required beyond 180-day period further formalities and documentation must be submitted[3]	August 2019
Vermont	No	Yes	-	Preferred buprenorphine products do not require PA but all non-preferred formulations require PA and quantity limit may apply[3]	October 2019
Virginia	No	Yes	-	Preferred buprenorphine products do not require PA but all non-preferred formulations require PA.[3]	October 2019
Washington	No	Yes	July 28, 2019	Medicaid managed care plan does not require PA on at least one FDA approved opioid agonists, antagonists, and partial agonists[22]	
West Virginia	No	Yes	-	PA may not be required for preferred buprenorphine formulation, but it is required for non-preferred formulations	October 2019
Wisconsin	No	Yes	November, 2018	PA is required for certain buprenorphine containing formulations[16]	May 2020

PA- prior authorizations, PDL-preferred drug list, OUD- opioid use disorder, MAT- Medication Assisted Treatments

© 2022 Keshwani S et al. *JAMA Health Forum.*

eTable 4. Analytical Dataset. Source: State Drug Utilization Data

Year, quarter	California		Illinois		Control States	
	Numerator	Denominator	Numerator	Denominator	Numerator	Denominator
2013,4	2976	9661152	3099	2934163	8411	6429386
2014,1	3824	10334000	2420	2791737	8750	6022549
2014,2	6547	10900000	3666	3003141	10117	6222496
2014,3	7969	11300000	2279	3098603	11859	6375878
2014,4	8885	11919314	2605	3126814	13422	6374089
2015,1	9796	12248555	2183	3198477	13520	6488865
2015,2	12366	12627234	1799	3163838	14123	6546112
2015,3	15628	11463750	3802	3147955	14535	6637983
2015,4	17752	12166109	8904	3134109	15629	6626559
2016,1	19268	11869623	9272	3150343	15945	6680437
2016,2	18432	12333253	11003	3123639	17576	6703785
2016,3	26822	12281044	11266	3116270	17980	7433234
2016,4	24948	12405352	18799	3065331	19712	7428542
2017,1	26536	12376741	24388	3097621	19735	7431779
2017,2	24632	12293428	20954	3075710	23119	6911107
2017,3	36104	12268887	22309	3070815	22384	6843013
2017,4	33593	12220546	22803	3062268	23019	6854673
2018,1	35814	12193802	24343	2978713	23775	6811950
2018,2	32470	12064332	23857	2989709	25897	6721252
2018,3	46957	12011621	24451	2955094	26845	6732842
2018,4	46424	11927676	25638	2860188	28567	6705386
2019,1	40934	11874143	26543	2800206	29148	6643936
2019,2	40106	11744007	28374	2826169	31354	6625351
2019,3	59716	11728031	29145	2842807	31493	6620451
2019,4	51210	11588323	30475	2812371	32286	6584936
2020,1	52934	11576877	30738	2855246	36130	6640203

© 2022 Keshwani S et al. *JAMA Health Forum.*

eMethods. Sensitivity Analyses: Synthetic Control Method

We conducted additional sensitivity analysis using the synthetic control method (SCM) to create the counterfactual rate outcome for California and Illinois. This synthetic control is a weighted average of the control states that best emulates the pre-policy trend in the intervention state.[23] The synthetic version of California and Illinois was constructed using a convex combination of states that had strict PA policies related to buprenorphine (Alabama, Florida, Idaho, Kansas, Mississippi, Nevada, South Dakota, and Wyoming) referred as donor pool.

Variable	Definition	Source
Pre-treatment outcomes	State buprenorphine prescribing rates for 2013.Q4, 2014.Q3 and 2015, Q2	State Drug Utilization Data
Opioid Prescribing	State opioid dispensing rate per 100 population (Averaged for 2014 and 2015)	CDC Injury Center [24,25]
Overdose deaths	State annual overdose deaths per 100 population (Averaged for 2014 and 2015)	CDC Injury Center[26,27]
Opioid Treatment Programs	State number of substance use disorder treatment facilities accepting Medicaid patients per 100 Medicaid enrollees (Averaged for 2014 and 2015)	SAMHSA. Data on Substance Abuse Treatment Facilities[28,29]

eFigure 1 and eFigure 2 shows the contributing weights of the donor states, pre-treatment outcomes and covariates for California and Illinois. We used tidysynth package in R for this analysis.[30]

Results

We estimated that by the end of our study period (i.e., 2020. Q1) the buprenorphine prescription rate in the Actual California Medicaid program was 0.45 per 100 Medicaid enrollees and for the Synthetic California was 0.433 per 100 Medicaid enrollees (eFigure 3). This represents a small increase in prescription rate by 0.02% than what would have been in the absence of removal of PA. (eFigure 4. In Illinois state Medicaid program, the buprenorphine prescription rate in the Actual Illinois was 1.07 per 100 Medicaid enrollees and for the Synthetic Illinois was 0.39 per 100 Medicaid enrollees (eFigure 5). This represents an increase in prescription rate by 0.68% than what would have been in the absence of removal of PA (eFigure 6).

© 2022 Keshwani S et al. *JAMA Health Forum.*

In this analysis using weighted control we observed a small increase in the buprenorphine prescription rate for California compared to its synthetic counterfactual. These results are not contradictory to our primary analysis though as we see in eFigure3 how the rate of prescribing in California closely overlaps that of the synthetic California trend. It is to be noted that the synthetic control method is an extension of difference-in-difference which does not report level and trend changes described in our primary analysis.[23]

Placebo analysis

We calculated the ratio of the mean squared prediction error (MSPE) between the post-treatment and pre-treatment periods using the synthetic counterfactuals for each state. We then ranked the MSPE ratios in descending order and constructed the p-value as rank/total number of states. For a meaning change due to the intervention (i.e., removal of PA related to buprenorphine) the synthetic control must have low MSPE in the pre-intervention period and high MSPE in the post-intervention period. A ratio close to one indicates that the intervention had no change. The MSPE ratio for California was 14.4 and for Illinois was 59.6 (eFigure 7, eFigure 8). This indicates that the post-treatment error is larger than the pre-treatment error.

© 2022 Keshwani S et al. *JAMA Health Forum.*

eFigure 1. States Weights and Variable Weights in the Synthetic California

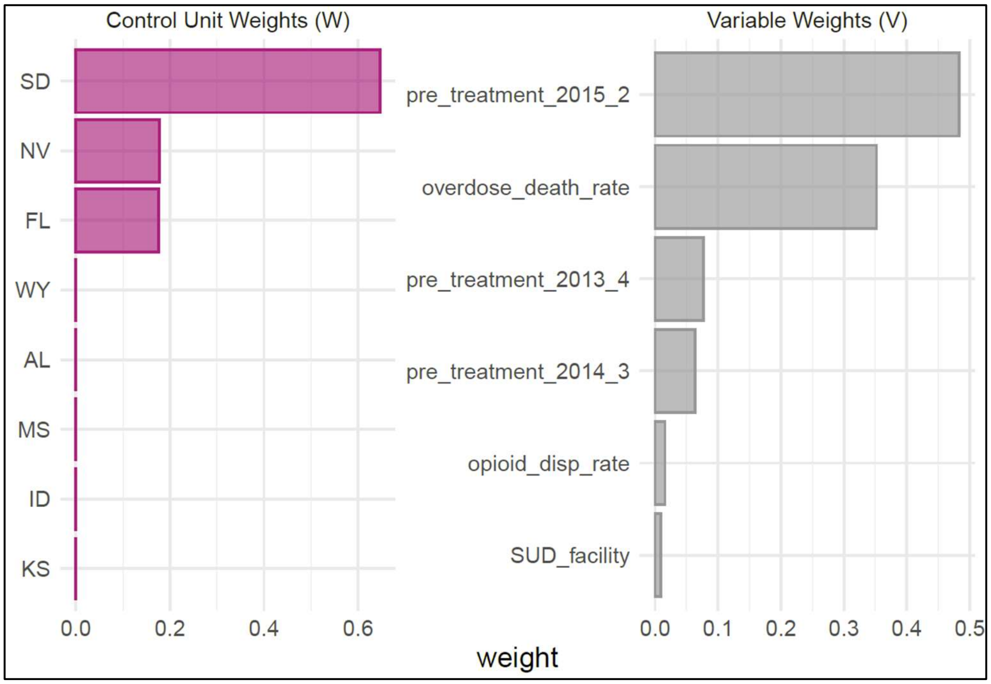

eFigure 2. States Weights and Variable Weights in the Synthetic Illinois

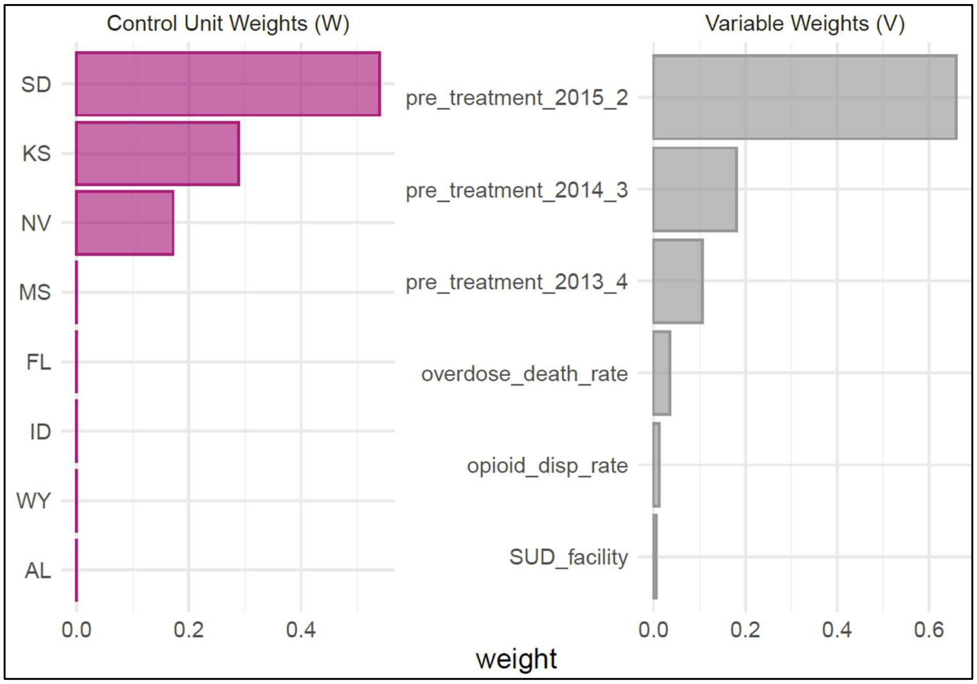

© 2022 Keshwani S et al. *JAMA Health Forum.*

eFigure 3. Trends in Buprenorphine Prescribing Rate in California vs. Synthetic California

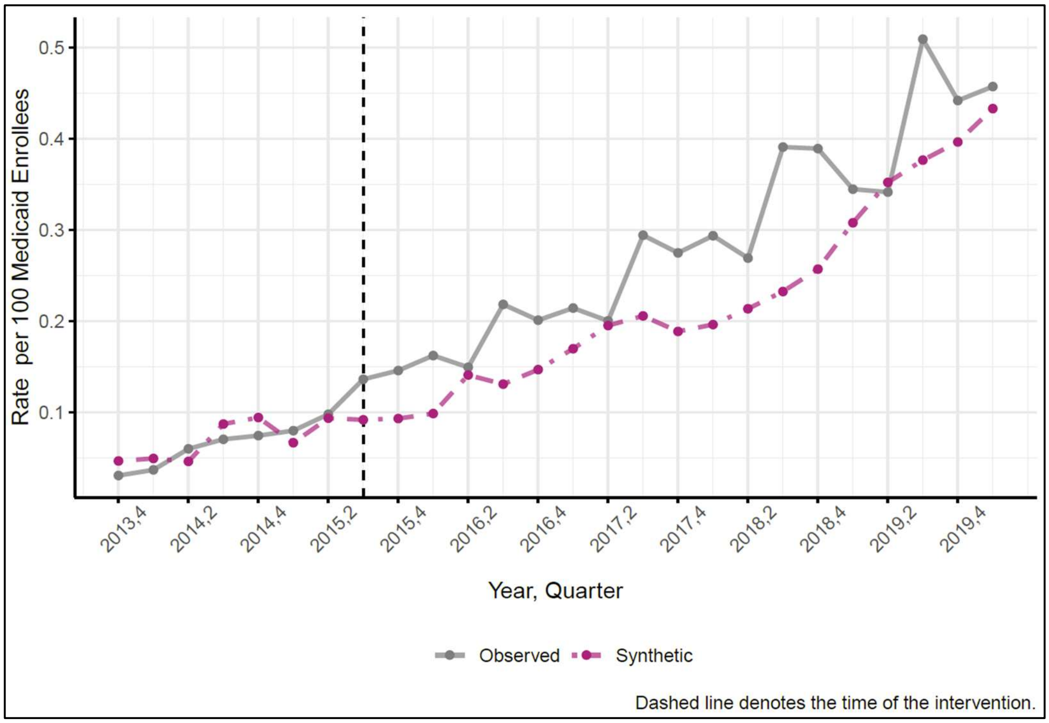

eFigure 4. Buprenorphine Prescribing Rate Gap in California vs. Synthetic California

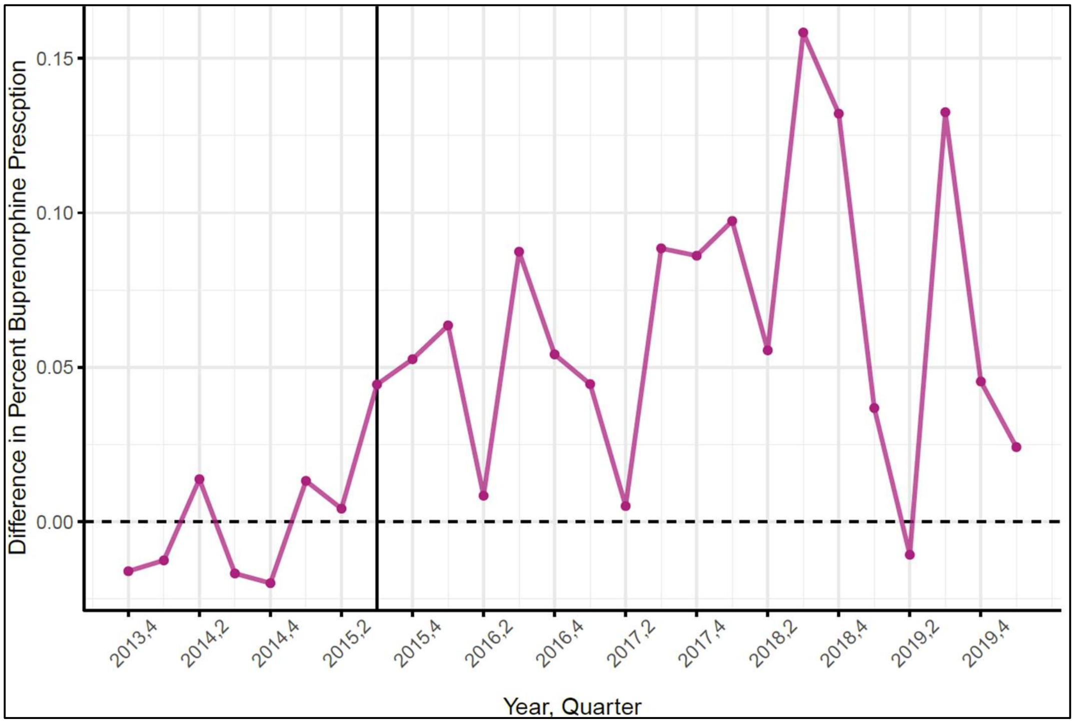

© 2022 Keshwani S et al. *JAMA Health Forum.*

eFigure 5. Trends in Buprenorphine Prescribing Rate in Illinois vs. Synthetic Illinois

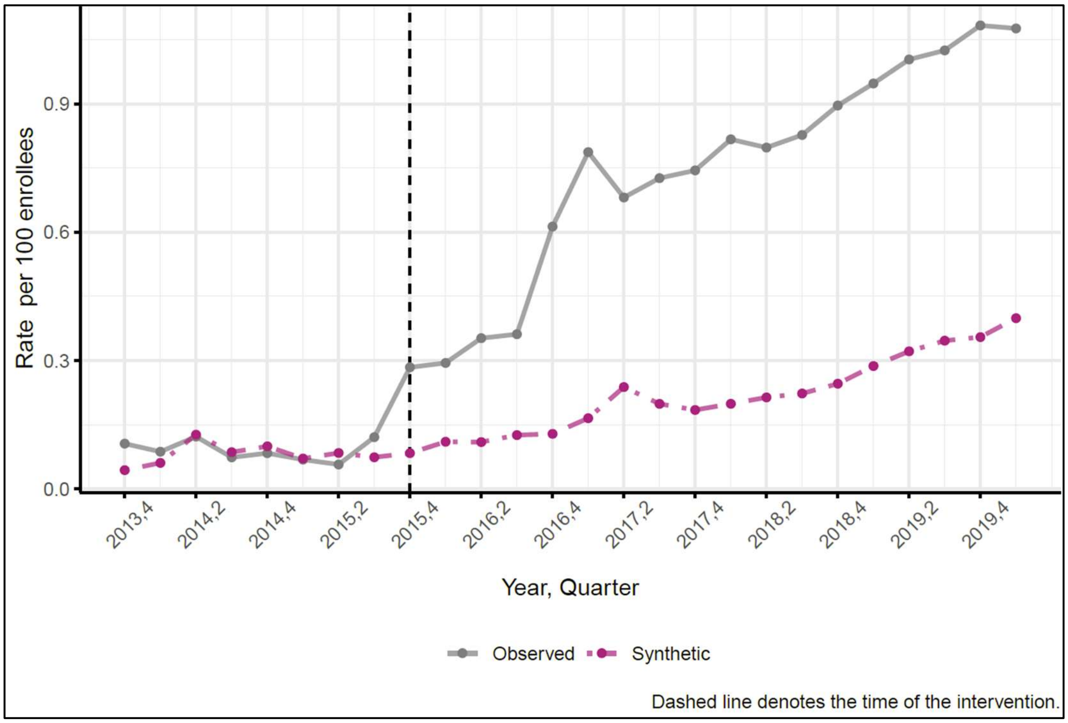

eFigure 6. Buprenorphine Prescribing Rate Gap in Illinois vs. Synthetic Illinois

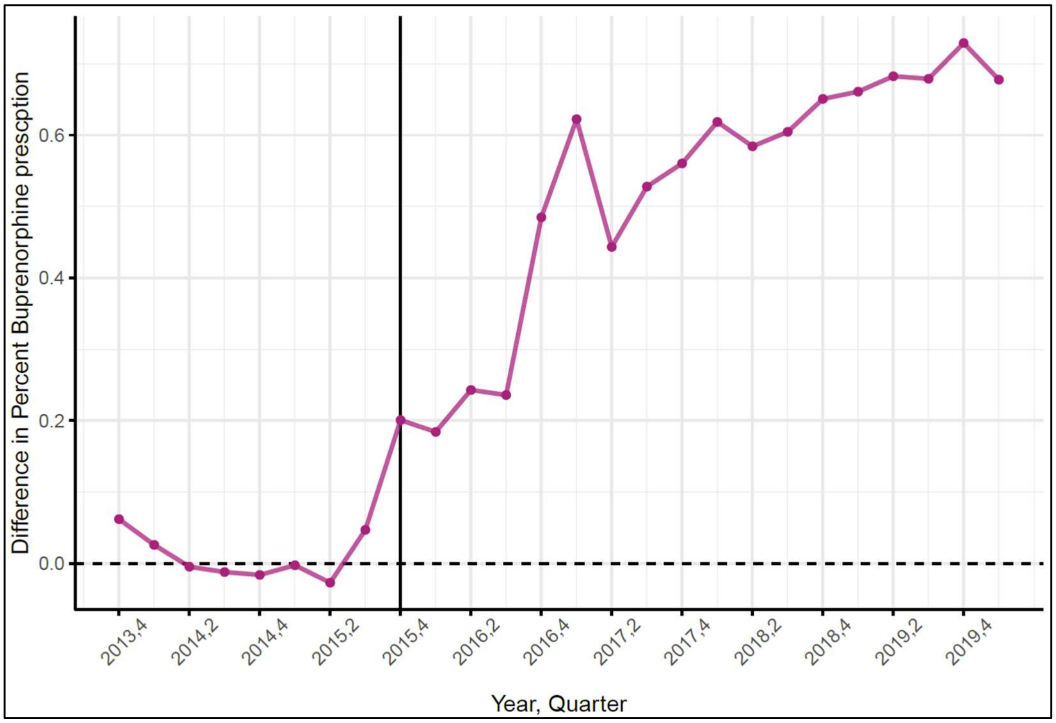

© 2022 Keshwani S et al. *JAMA Health Forum.*

eFigure 7. Ratio of Post-Intervention RMSPE to Pre-Intervention RMSPE: California and Control States

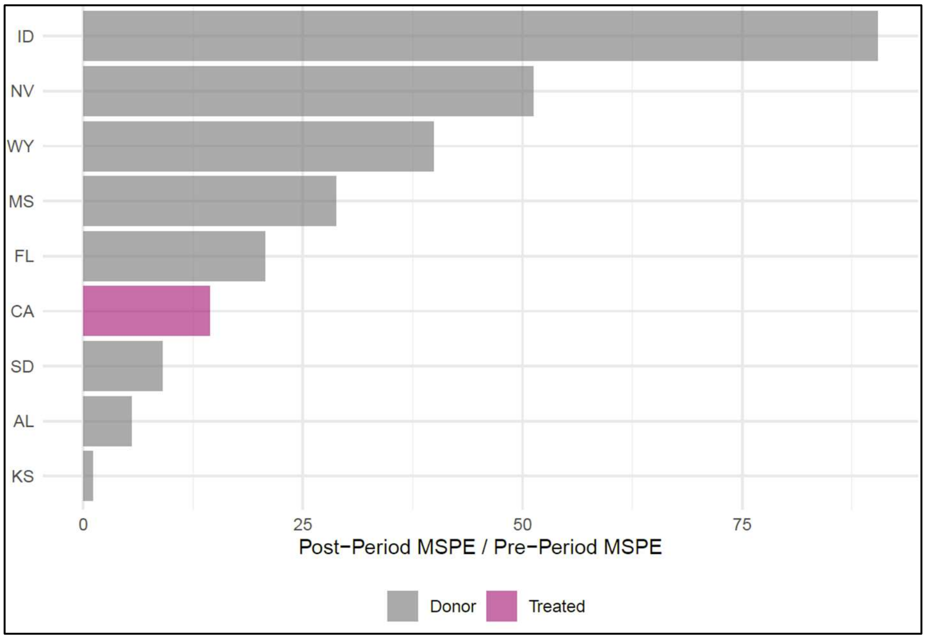

RMSPE: Root Mean Square Prediction Error

eFigure 8. Ratio of Post-Intervention RMSPE to Pre-Intervention RMSPE: Illinois and Control States

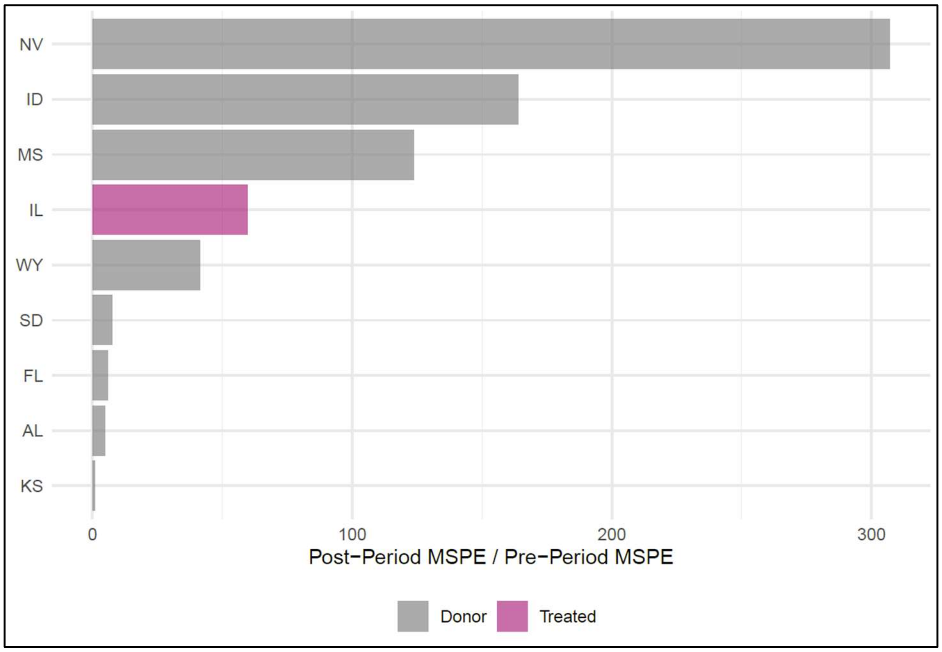

RMSPE: Root Mean Square Prediction Error

© 2022 Keshwani S et al. *JAMA Health Forum.*

eReferences

1. Medi-Cal. Clinical Review: The Treatment of Opioid Addiction with Buprenorphine. https://files.medi-cal.ca.gov/pubsdoco/dur/Articles/dured_25096.pdf. Published August 31, 2016. Accessed June 4, 2021.
2. Rowan HB. Use Of Buprenorphine To Treat Opioid Addiction Proliferates In California. https://khn.org/news/use-of-buprenorphine-to-treat-opioid-addiction-proliferates-in-california/. Published May 24, 2019. Accessed June 4, 2021.
3. Miller N. Comprehensive Update on State Medicaid Coverage of Medication Assisted Treatments and Substance Use Disorder Services. *Advocatesfor Human Potential, Inc*. December 2019. https://www.rsat-tta.com/Files/MAT-Medicaid-Review_updated-12-2019. Accessed June 1, 2021.
4. 305 ILCS 5/5-5 Medical services.,. *305 ILCS 5/5-5 (Statutes current with legislation through P.A. 102-15 of the 2021 Session of the 102nd Legislature.)*. https://advance.lexis.com/api/document?collection=statutes-legislation&id=urn:contentItem:8W96-TS22-D6RV-H359-00000-00&context=1516831. Accessed July 15, 2021.
5. Alabama's Oversight of Opioid Prescribingand Monitoring of Opioid Use. https://oig.hhs.gov/oas/reports/region4/41900125_Factsheet.pdf. Published November 2019. Accessed March 5, 2022.
6. Idaho Medicaid. Idaho Medicaid Preferred Drug List with Prior Authorization Criteria. https://publicdocuments.dhw.idaho.gov/WebLink/DocView.aspx?id=15075&dbid=0&repo=PUBLIC-DOCUMENTS&cr=1. Published January 2022. Accessed March 5, 2022.
7. Nevada Medicaid. Informational Bulletin on Medications and Services for Substance Use Disorders. https://dhcfp.nv.gov/uploadedFiles/dhcfpnvgov/content/Pgms/CPT/NewsletterDrugCoverage06-2016V2.pdf. Accessed March 5, 2022.
8. Department of Health and Social Services. Alaska Medicaid Pharmacy Opioid Management Strategies and Initiatives – 2017. https://alaskapharmacy.org/wp-content/uploads/2017/06/DUR_OpioidManagementStrategies_2017.pdf. Published March 21, 2017. Accessed March 6, 2022.
9. Arkansas Code-Title 23-Subtitle 3-Chapter 99-Subchapter 11. Prior Authorization Transparency Act - Managed Care Contracting. https://managedcarelegaldatabase.org/state-law/title-23-subtitle-3-chapter-99-healthcare-providers-subchapter-11-prior-authorization-transparency-act/. Accessed March 7, 2022.
10. HB 19-1269 - Colorado House (2019A) - Open States. https://openstates.org/co/bills/2019A/HB19-1269/. Accessed March 8, 2022.
11. Delaware Health and Social Services Division of Medicaid and Medical Assistance. Delaware Medical Assistance Program (DMAP) Preferred Drug List (PDL). https://eforms.com/images/2017/05/Delaware-Medicaid-Preferred-Drug-List.pdf. Published April 28, 2017. Accessed June 3, 2021.
12. Government of District Columbia. Removal of Prior Authorization Requirements for Medication Assisted- Treatment. https://dhcf.dc.gov/sites/default/files/dc/sites/dhcf/publication/attachments/Policy%20%2319-001%20Removal%20of%20Prior%20Auth.%20Req.%20for%20Medication-Assis_0.pdf. Accessed March 8, 2022.
13. Griffin G, McGuire L. *Opioid Use Disorder – Access to Medication-Assisted Treatment* . Georgia Department of Audits and Accounts Performance Audit Division; 2017. http://file:///C:/Users/shail/OneDrive/Desktop/17-11%20MAT%20Access%20-%20Amended.pdf. Accessed June 25, 2021.
14. Iowa Legislature. HF623. https://www.legis.iowa.gov/legislation/BillBook?ga=88&ba=HF623. Published January 5, 2019. Accessed March 7, 2022.
15. Louisiana Department of Health. Prior Authorizations for Medications Used to Treat Opioid Use Disorder. https://ldh.la.gov/assets/docs/LegisReports/HR257RS201912020.pdf#:~:text=HR%20257%20of%20the%202019%20regular%20legislative%20session,used%20for%20the%20treatment%20of%20opioid%20use%20disorder. Published January 2020. Accessed March 8, 2022.

© 2022 Keshwani S et al. *JAMA Health Forum.*

16. Weber E. Spotlight on Legislation Limiting the Use of Prior Authorization for Substance Use Disorder Services and Medications. *LAC*. May 2020. https://www.lac.org/resource/spotlight-on-legislation-limiting-the-use-of-prior-authorization-for-substance-use-disorder-services-and-medications. Accessed April 19, 2021.
17. New Hampshire Healthy Familie. Formulary New Hampshire Healthy Families. https://pharmacy.envolvehealth.com/content/dam/centene/envolve-pharmacy-solutions/pdfs/PDL/FORMULARY-NewHampshireHealthyFamilies.pdf. Published March 1, 2022. Accessed March 8, 2022.
18. Legiscan. New Jersey Assembly Bill 4744. https://legiscan.com/NJ/text/A4744/2018. Accessed March 8, 2022.
19. Oklahoma Health Care Authority. Substance Use Stewardship. https://oklahoma.gov/content/dam/ok/en/okhca/documents/a0302/23638.pdf. Accessed March 8, 2022.
20. BillTrack50. OR HB2257 . https://www.billtrack50.com/BillDetail/1011831#:~:text=bill%20summary,-Chamber&text=H-,Chapter%20583%2C%20(2019%20Laws)%3A,Effective%20date%20July%2023%2C%202019.&text=Governor%20signed. Published 2019. Accessed March 8, 2022.
21. Division of TenneCare. Office-based Buprenorphine Treatment. https://www.tn.gov/tenncare/tenncare-s-opioid-strategy/office-based-buprenorphine-treatment.html. Published 2019. Accessed March 9, 2022.
22. Washington State Legislature. RCW 74.09.645: Opioid use disorder—Coverage without prior authorization. https://app.leg.wa.gov/RCW/default.aspx?cite=74.09.645. Published 2019. Accessed March 9, 2022.
23. Abadie A, Diamond A, Hainmueller J. Synthetic Control Methods for Comparative Case Studies: Estimating the Effect of California's Tobacco Control Program. *J Am Stat Assoc*. 2010;105(490):493-505. doi:10.1198/jasa.2009.ap08746
24. CDC Injury Center. U.S. State Opioid Dispensing Rates, 2014 . https://www.cdc.gov/drugoverdose/rxrate-maps/state2014.html. Accessed April 27, 2022.
25. CDC Injury Center. U.S. State Opioid Dispensing Rates, 2015. https://www.cdc.gov/drugoverdose/rxrate-maps/state2015.html. Accessed April 27, 2022.
26. CDC Injury Center. 2014 Drug Overdose Death Rates . https://www.cdc.gov/drugoverdose/deaths/2014.html. Accessed April 27, 2022.
27. CDC Injury Center. 2015 Drug Overdose Death Rates. https://www.cdc.gov/drugoverdose/deaths/2015.html. Accessed April 27, 2022.
28. SAMHSA. 2014 Data on Substance Abuse Treatment Facilities. https://www.samhsa.gov/data/sites/default/files/2014_National_Survey_of_Substance_Abuse_Treatment_Services/2014_National_Survey_of_Substance_Abuse_Treatment_Services/2014_National_Survey_of_Substance_Abuse_Treatment_Services.pdf. Published March 2016. Accessed April 27, 2022.
29. SAMHSA. 2015 Data on Substance Abuse Treatment Facilities. https://www.samhsa.gov/data/sites/default/files/2015_National_Survey_of_Substance_Abuse_Treatment_Services.pdf. Published March 2017. Accessed April 27, 2022.
30. E D. A tidy implementation of the synthetic control method in R. https://github.com/edunford/tidysynth. https://github.com/edunford/tidysynth. Published 2021. Accessed April 26, 2022.

© 2022 Keshwani S et al. *JAMA Health Forum.*
